# Impact of breast cancer risk factors on clinically relevant prognostic biomarkers for primary breast cancer

**DOI:** 10.1007/s10549-021-06294-5

**Published:** 2021-06-29

**Authors:** Mustapha Abubakar, Changyuan Guo, Hela Koka, Bin Zhu, Joseph Deng, Nan Hu, Bin Zhou, Montserrat Garcia-Closas, Ning Lu, Xiaohong R. Yang

**Affiliations:** 1 Division of Cancer Epidemiology and Genetics, National Cancer Institute, National Institutes of Health (NIH), Bethesda, USA; 2 National Cancer Center/Cancer Hospital, Chinese Academy of Medical Sciences and Peking Union Medical College, Beijing, 100021 China

**Keywords:** Age, Parity, Overweight, Obese, Family history, Breast cancer, Prognosis, NPI, IHC4

## Abstract

**Purpose:**

In addition to impacting incidence, risk factors for breast cancer may also influence recurrence and survival from the disease. However, it is unclear how these factors affect combinatorial biomarkers for aiding treatment decision-making in breast cancer.

**Methods:**

Patients were 8179 women with histologically confirmed invasive breast cancer, diagnosed and treated in a large cancer hospital in Beijing, China. Individual clinicopathological (tumor size, grade, lymph nodes) and immunohistochemical (IHC: ER, PR, HER2, KI67) markers were used to define clinically relevant combinatorial prognostic biomarkers, including the Nottingham Prognostic Index (NPI: combining size, grade, nodes) and IHC4 score (combining ER, PR, HER2, KI67). Odds ratios (ORs) and 95% confidence intervals (CIs) for associations between breast cancer risk factors and quartiles (Q1–Q4) of NPI and IHC4 were assessed in multivariable polytomous logistic regression models.

**Results:**

Overall, increasing parity (OR_trend_(95% CI) = 1.20(1.05–1.37);*P*_trend_ = 0.007), overweight (OR(95% CI)_vs normal_ = 1.60(1.29–1.98)), and obesity (OR(95% CI) _vs normal_ = 2.12(1.43–3.14)) were associated with higher likelihood of developing tumors with high (Q4) versus low (Q1) NPI score. Conversely, increasing age (OR_trend_(95% CI) = 0.75(0.66–0.84);*P*_trend_ < 0.001) and positive family history of breast cancer (FHBC) (OR(95% CI) = 0.66(0.45–0.95)) were inversely associated with NPI. Only body mass index (BMI) was associated with IHC4, with overweight (OR(95% CI) _vs normal_ = 0.82(0.66–1.02)) and obese (OR(95% CI) _vs normal_ = 0.52(0.36–0.76)) women less likely to develop high IHC4 tumors. Notably, elevated BMI was associated with higher NPI irrespective of hormone receptor-expression status.

**Conclusions:**

Our findings indicate that factors affecting breast cancer incidence, particularly age, parity, FHBC, and BMI, may impact clinically relevant prognostic biomarkers with implications for surveillance, prognostication, and counseling.

**Supplementary Information:**

The online version contains supplementary material available at 10.1007/s10549-021-06294-5.

## Background

Breast cancer is a clinically heterogeneous disease and differences in tumor behavior can influence treatment recommendations and clinical outcomes, including recurrence and survival, among breast cancer patients [1, 2]. In addition to influencing breast cancer incidence, risk factors such as elevated body mass index (BMI), nulliparity, lack of breastfeeding, and older age at first full-term pregnancy may also influence clinical outcomes following breast cancer diagnosis [3–8]. However, it is unclear whether these factors affect combinatorial biomarkers of breast cancer prognosis, which aid to guide treatment selection and patient management.

In general, most previous studies examining the relationships between breast cancer risk factors and tumor behavior have focused on the individual tumor characteristics, and not their constellation. Higher BMI, for instance, is reportedly associated with higher grade or larger size tumors; nulliparity and menopausal hormone therapy use (MHT) with highly proliferating and lobular carcinomas, respectively; and higher parity with P53 expressing tumors [9–12]. The impact of these factors on prognostic biomarkers that combine several tumor characteristics to infer tumor aggressiveness and aid treatment recommendation is, however, less well-studied.

To aid prognostication in breast cancer, several tumor characteristics including histologic grade, tumor size, lymph nodal involvement, estrogen receptor (ER), progesterone receptor (PR), human epidermal growth factor receptor 2 (HER2) and KI67, a marker of proliferation, have been combined into clinically useful prognostic algorithms [13–16]. The Nottingham prognostic index (NPI) is popular for combining information on tumor size, histologic grade, and lymph nodal involvement into a single quantitative measure [13]. The IHC4 score, on the other hand, combines information on ER, PR, HER2, and KI67 into a prognostic algorithm [16].

Despite their prognostic relevance, it remains unclear whether breast cancer risk factors influence these combinatorial prognostic markers. Our main aim in this study was, therefore, to investigate the associations between breast cancer risk factors and breast tumor behavior defined by NPI and IHC4 score among a cohort of Chinese breast cancer patients. Key findings from the primary analysis were then re-evaluated in an independent cohort of Polish patients.

## Methods and materials

### Study population

The main study population comprised Chinese breast cancer patients from a hospital-based case series who had histologically confirmed invasive breast cancer that were diagnosed and treated at the Cancer Hospital, Chinese Academy of Medical Sciences (CHCAMS), Beijing, China. Overall, a total of 8616 patients, aged 29–97 years at diagnosis, were recruited from CHCAMS between 2011 and 2016. Of these, 437 patients did not have complete data on hormone receptor status, i.e. ER and/or PR and hence were excluded from the analysis. This study received ethical approval from the CHCAMS Ethics Committee. Owing to the fact that this analysis did not involve interaction with human subjects or the use of individual’s personal identifying information, it was granted exemption from review by the Office of Human Research Protections at the National Institutes of Health, NIH (exempt number 11751). The Polish Breast Cancer Study (PBCS) is a population-based study in Poland that enrolled women 20–74 years with histologically or cytologically confirmed breast cancer (*n* = 2386) at five participating hospitals in Warsaw and Lodz over a three-year period between 2000 and 2003 [17]. For the current analysis, we identified 972 patients from the PBCS study with complete information to allow the generation of the IHC4 score and/or NPI. Ethical approvals for PBCS were obtained from local ethics committees and all participants provided written informed consent as required by local institutional and National Cancer Institute/NIH review boards.

### Data on tumor clinicopathological features and breast cancer risk factors

Data on tumor clinicopathological features, including morphology, histologic grade, tumor size, lymph nodal involvement, ER, PR, HER2, and KI67 were obtained from pathology records. Data on breast cancer risk factors, including age, BMI, family history of breast cancer (FHBC) in a first degree relative, as well as reproductive factors, including age at menarche, parity and number of children, and breastfeeding were extracted from patients’ medical records. These data were collected from patients and entered into the medical records as part of patients’ medical workup. Anthropometric measures, including height and weight, were obtained by trained members of staff during clinical workup.

### Immunohistochemical staining

Details of IHC staining and scoring procedures have been previously described [18]. In brief, all IHC markers were stained using standard laboratory procedures. ER, PR, and HER2 were stained using Roche rabbit monoclonal antibodies, SP1, 1E2, and 4B5 clones, at 1:1000, 1:1000, and 1:66 dilutions, respectively. KI67 was stained using mouse monoclonal antibody MIB1 based on the manufacturer optimized concentration. Staining was performed using the Roche Ventana XT autostainer for all markers. Scoring was performed by pathologists with expertise in breast cancer. Based on international conventions [2], ER and PR positivity were defined as >1% positively staining cells while for HER2, 3 + on IHC or amplification on fluorescent in situ hybridization (FISH) was considered positive. In keeping with results from a previous meta-analysis showing KI67 score of 25% to provide the best survival discrimination [19], we dichotomized KI67 at this cutoff-point with scores of >25% designating high KI67 expression. Binary categories of KI67 were used in combination with standard clinical categories of ER, PR, and HER2 to define breast cancer subtypes (luminal A-like, luminal B-like, HER2-enriched, and triple negative breast cancer [TNBC]) according to internationally recommended guidelines [20]. Continuous measures of ER, PR, and KI67 were used in combination with HER2 for the calculation of the IHC4 score in keeping with the validated algorithm [16].

### Computation of the NPI and IHC4 score

We calculated the NPI based on the published equation combining tumor size, grade, and nodal involvement [13]:

- **NPI** = 0.2 × tumor size (cm) + grade + nodal status (pN0 = 1, pN1-3 = 2, pN ≥ 4 = 3)

We calculated the IHC4 score based on the published equation combining ER, PR, HER2 and KI67 [16]:

- **IHC4 score** = 94.7 × {(−0.100ER10) + (−0.079PR10) + (0.586HER2) + [0.240ln(1+10×Ki67)]}

### Statistical analysis

The following breast cancer risk factors were examined: age: <35 (reference), 35–45, 45–55, >55 years; parity: nulliparous (reference), 1, 2, ≥3 children; age at menarche: ≤12 (reference), 13, 14, ≥15 years; BMI, in kg/m^2^: underweight (<18.5), normal (18.5–25; reference), overweight (25–30), and obese (>30); and FHBC in a first degree relative: yes (if present) or no (if absent; reference). NPI and IHC4 score were categorized into quartiles as follows; Q1 (<25th percentile); Q2 (25th–50th percentile); Q3 (50th–75th percentile); and Q4 (>75th percentile). Associations between breast cancer risk factors and tumor-related prognostic indicators were assessed in polytomous logistic regression models with quartiles of NPI and IHC4 score as outcomes (Q1 = baseline category) and breast cancer risk factors as predictors. Analyses were performed overall and following stratification by age (≤50 and >50), as surrogate for menopausal status, and by tumor hormone receptor (HR) expression status (i.e. HR + and HR−). In sensitivity analysis, we used PREDICT[14], another combinatorial prognostic marker that contains tumor size, grade, and lymph nodal involvement, in place of the NPI and assessed relationships with breast cancer risk factors. To assess which if any, of the individual clinicopathologic (tumor size, histologic grade, nodal involvement) and IHC (ER, PR, HER2 and KI67) factors was driving associations between risk factors and these combinatorial prognostic factors, we modeled relevant breast cancer risk factors as outcome variables and mutually adjusted for the individual clinicopathological and IHC features as predictors. The contribution of individual factors to model prediction was determined by assessing change in likelihood ratio Chi-square (LRχ^2^) when the factor was removed from the fully adjusted model. For those factors that were significantly related to NPI and/or IHC4 in the Chinese population, we repeated the analysis in an independent sample of 972 breast cancer cases from PBCS [17]. Missing values on risk factor covariates were addressed by the listwise deletion approach. In additional sensitivity analyses, we created indicators for missing values on the covariates that were included in multivariable models. All analyses were two-sided and performed using Stata statistical software version 16.1.

## Results

### Distribution of baseline clinicopathological factors and breast cancer risk factors among study participants

Table 1 describes the distribution of clinicopathologic as well as lifestyle and reproductive factors in this dataset consisting of 8,179 breast cancer patients, overall and stratified by HR status. The mean age at diagnosis was 51.8 years, which did not differ significantly by HR status. The majority of these patients had intermediate or high grade (91%) tumors. Similarly, node negative (53%), ER + (77%), PR + (76%), HER2- (80%), and low KI67 (55%) tumors predominated. Notably, the frequencies of all clinicopathologic characteristics differed significantly by HR status, with HR– tumors having higher frequencies of aggressive tumor characteristics than HR + tumors. In terms of breast cancer risk factors, the majority of the patients had at least one child (97%), had breastfed (87%), had normal BMI (54%), and had a negative FHBC in a first degree relative (92%), none of which varied by HR status (Table 1). In general, patients from the PBCS study were older, experienced menarche earlier, and were more frequently nulliparous or obese than patients from the CHCAMS study (Supplementary Table 1).

**Table 1:** Distributions of clinicopathological and breast cancer risk factors, overall and by tumor hormone receptor expression status, among Chinese breast cancer patients

Characteristic	Overall (*n* = 8179)		HR + (*n* = 6675)		HR− (*n* = 1504)		*P value*
	Freq	%	Freq	%	Freq	%	
Age, years							
<35	454	5.3	347	5.2	75	5.0	
35–45	2095	24.3	1640	24.5	335	22.3	
45–55	2982	34.7	2301	34.5	544	36.1	
>55	3068	35.7	2387	35.8	550	36.6	0.26
Histologic grade							
Low	670	8.6	624	10.2	11	0.8	
Intermediate	4655	59.9	4051	66.1	412	30.6	
High	2450	31.5	1455	23.7	923	68.6	<0.001
Size							
≤2 cm	3531	47.3	2799	48.0	540	40.6	
>2 cm	3939	52.7	3027	52.0	791	59.4	<0.001
Nodal involvement							
None	3798	53.0	2968	52.2	713	57.1	
1	985	13.8	795	14.0	153	12.3	
2	563	7.9	469	8.3	79	6.3	
≥3	1806	25.3	1451	25.5	303	24.3	0.006
ER							
Negative	1897	23.2					
Positive	6285	76.8					
PR							
Negative	1952	23.9					
Positive	6221	76.1					
HER2							
Negative	6556	80.3	5627	84.5	926	61.8	
Positive	1604	19.7	1031	15.5	572	38.2	<0.001
KI67							
Low	4432	55.2	4066	62.0	356	24.5	
High	3598	44.8	2494	38.0	1098	75.5	<0.001
Age at Menarche, years							
≤12	730	10.9	596	11.2	106	9.1	
13	1248	18.6	982	18.4	235	20.2	
14	1509	22.6	1197	22.4	354	21.9	
≥15	3207	47.9	2560	48.0	566	48.8	0.13
Parity							
Nulliparous	304	3.5	241	3.6	45	3.0	
1	3985	46.3	3186	47.7	676	45.0	
2	1920	22.3	1497	22.4	354	23.5	
≥3	2407	27.9	1751	26.3	429	28.5	0.09
Breastfeeding							
Never	733	12.5	584	12.6	111	10.5	
Ever	5142	87.5	4045	87.4	945	89.5	0.06
Body mass index (BMI)							
Underweight	155	2.1	121	2.1	25	2.0	
Normal	3946	54.2	3090	53.7	703	55.4	
Overweight	2586	35.5	2063	35.9	440	34.6	
Obese	595	8.2	480	8.3	102	8.0	0.76
Family history							
Absent	6721	92.4	5316	92.4	1180	92.8	
Present	554	7.6	440	7.6	91	7.2	0.55

^*^P values were obtained from Chi-squared tests for categorical variables

### Associations between breast cancer risk factors and levels of Nottingham prognostic index (NPI)

Table 2 shows the associations between breast cancer risk factors and the NPI. We found increasing age to be statistically significantly inversely associated with higher NPI (OR_trend_ (95% CI) = 0.75 (0.66–0.84), P_trend_ < 0.001). Conversely, higher parity was significantly associated with higher NPI (OR_trend_ (95% CI) = 1.20 (1.05–1.37), P_trend_ = 0.007). Furthermore, overweight (OR (95% CI) = 1.60 (1.29–1.98)) and obese (OR (95% CI) = 2.12 (1.43–3.14) women were substantially more likely than normal weight women to have high (Q4 *vs* Q1) NPI values. There was a significant trend with increasing BMI and NPI (OR_trend_ (95% CI) = 1.53 (1.30–1.79), P_trend_ < 0.001). A positive FHBC in a first degree relative was inversely associated with higher NPI values (OR (95% CI) _Q4 vs Q1_ = 0.66 (0.45–0.95); *P* = 0.027). Other factors, including breastfeeding and age at menarche were not associated with the NPI. In sensitivity analysis using PREDICT, we found strikingly similar associations between age, parity, BMI, and family history with PREDICT as we did in relation to the NPI (Supplementary Table 2). The associations between parity, BMI, and FHBC with NPI were similar in women with HR + and HR− breast cancer (Table 3), in younger and older women (Supplementary Table 3), and in analysis accounting for missing values on covariates (Supplementary Table 4).

**Table 2:** Odds ratios (ORs) and 95% confidence intervals (CIs) for the associations between breast cancer risk factors and levels of the Nottingham prognostic index (NPI) among Chinese breast cancer patients with complete data (*n* = 3668)

Characteristic	NPI						
	Q1 (reference)	Q2		Q3		Q4	
	N	N	OR (95% CI)	N	OR (95% CI)	N	OR (95% CI)
*Age, years*							
<35	26	43	1.00 (reference)	56	1.00 (reference)	74	1.00 (reference)
35–45	211	229	0.66 (0.38, 1.13)	234	0.49 (0.29, 0.84)	208	0.31 (0.18, 0.52)
45–55	322	326	0.54 (0.32, 0.92)	315	0.36 (0.21, 0.60)	294	0.23 (0.14, 0.39)
>55	326	387	0.64 (0.37, 1.10)	316	0.34 (0.20, 0.57)	301	0.22 (0.13, 0.37)
OR_trend_			0.94 (0.84, 1.06)		0.79 (0.71, 0.89)		0.75 (0.66, 0.84)
* P trend*			0.33		<0.001		<0.001
*Age at Menarche, years*							
≤12	105	103	1.00 (reference)	98	1.00 (reference)	100	1.00 (reference)
13	175	194	1.11 (0.77, 1.58)	179	1.02 (0.70, 1.47)	152	0.88 (0.60, 1.28)
14	206	241	1.18 (0.83, 1.66)	210	1.04 (0.73, 1.48)	198	0.98 (0.68, 1.40)
≥15	399	447	1.08 (0.78, 1.49)	434	1.06 (0.76, 1.48)	427	1.03 (0.74, 1.43)
OR_trend_			1.01 (0.92, 1.11)		1.02 (0.93, 1.13)		1.04 (0.94, 1.15)
* P* trend			0.85		0.64		0.40
*Parity*							
Nulliparous	37	47	1.00 (reference)	37	1.00 (reference)	28	1.00 (reference)
1	485	510	0.83 (0.52, 1.34)	425	0.95 (0.57, 1.59)	399	1.29 (0.74, 2.23)
2	219	263	0.81 (0.49, 1.33)	269	1.21 (0.71, 2.06)	251	1.60 (0.90, 2.84)
≥3	144	165	0.67 (0.40, 1.14)	190	1.18 (0.67, 2.06)	199	1.75 (0.97, 3.17)
OR_trend_			0.91 (0.80, 1.04)		1.13 (0.99, 1.29)		1.20 (1.05, 1.37)
* P* trend			0.17		0.07		0.007
*Breastfeeding*							
Never	103	106	1.00 (reference)	90	1.00 (reference)	89	1.00 (reference)
Ever	687	717	0.97 (0.71, 1.31)	691	1.16 (0.83, 1.60)	652	1.10 (0.79, 1.53)
* P value*			0.82		0.38		0.55
*BMI*							
Underweight	19	16	0.75 (0.38, 1.52)	15	0.76 (0.37, 1.57)	17	0.94 (0.47, 1.90)
Normal	537	508	1.00 (reference)	474	1.00 (reference)	433	1.00 (reference)
Overweight	278	375	1.47 (1.20, 1.81)	341	1.46 (1.18, 1.81)	345	1.60 (1.29, 1.98)
Obese	51	86	1.91 (1.30, 2.80)	91	2.27 (1.54, 3.33)	82	2.12 (1.43, 3.14)
OR_trend_			1.42 (1.22, 1.66)		1.50 (1.28, 1.76)		1.53 (1.30, 1.79)
* P trend*			<0.001		<0.001		<0.001
*Family history*							
Absent	792	913	1.00 (reference)	863	1.00 (reference)	822	1.00 (reference)
Present	93	72	0.73 (0.52, 1.03)	58	0.64 (0.45, 0.93)	55	0.66 (0.45, 0.95)
* P value*			0.07		0.02		0.03

ORs and 95% CIs were from polytomous logistic regression models (Q1 was the base (comparison) category) with mutual adjustments for age, parity, age at menarche, body mass index (BMI), and family history. Parity was adjusted in the main model (presented in table) and this was substituted for breastfeeding in a separate model. All models were further adjusted for year of diagnosis and breast cancer subtype

**Table 3:** Odds ratios (ORs) and 95% confidence intervals (CIs) for associations between parity, body mass index (BMI), and family history and levels of the Nottingham prognostic index (NPI) in stratified analyses by hormone receptor-expression status among Chinese breast cancer patients

Characteristic	NPI score		
	Q2 *vs* Q1	Q3 *vs* Q1	Q4 *vs* Q1
	OR (95% CI)	OR (95% CI)	OR (95% CI)
HR +			
* Parity*			
Nulliparous	1.00 (reference)	1.00 (reference)	1.00 (reference)
1	1.01 (0.61, 1.70)	1.04 (0.61, 1.76)	1.39 (0.78, 2.46)
2	0.90 (0.52, 1.54)	1.24 (0.72, 2.16)	1.71 (0.94, 3.11)
≥3	0.73 (0.41, 1.29)	1.18 (0.66, 2.11)	1.74 (0.94, 3.25)
* OR*_*trend*_	0.89 (0.77, 1.02)	1.10 (0.96, 1.27)	1.18 (1.02, 1.36)
* P trend*	0.09	0.18	0.02
* BMI*			
Underweight	0.75 (0.34, 1.65)	0.98 (0.47, 2.04)	0.94 (0.45, 2.00)
Normal	1.00 (reference)	1.00 (reference)	1.00 (reference)
Overweight	1.40 (1.12, 1.75)	1.54 (1.22, 1.93)	1.54 (1.23, 1.94)
Obese	1.88 (1.24, 2.83)	2.56 (1.72, 3.82)	2.08 (1.37, 3.15)
* OR*_*trend*_	1.39 (1.17, 1.64)	1.59 (1.34, 1.87)	1.49 (1.26, 1.77)
* P trend*	<0.001	<0.001	<0.001
* Family history*			
Absent	1.00 (reference)	1.00 (reference)	1.00 (reference)
Present	0.75 (0.52, 1.08)	0.58 (0.39, 0.86)	0.64 (0.43, 0.95)
* P value*	0.11	0.007	0.02
HR−			
* Parity*			
Nulliparous	1.00 (reference)	1.00 (reference)	1.00 (reference)
1	0.40 (0.12, 1.32)	1.17 (0.27, 5.10)	0.92 (0.22, 3.73)
2	0.48 (0.14, 1.65)	1.23 (0.27, 5.59)	0.90 (0.21, 3.82)
≥3	0.60 (0.16, 2.18)	1.60 (0.33, 7.74)	1.55 (0.35, 6.92)
* OR*_*trend*_	1.07 (0.79, 1.43)	1.10 (0.80, 1.52)	1.17 (0.85, 1.59)
* P trend*	0.67	0.55	0.33
* BMI*			
Underweight	1.22 (0.19, 7.68)	1.08 (0.14, 8.07)	2.03 (0.32, 12.94)
Normal	1.00 (reference)	1.00 (reference)	1.00 (reference)
Overweight	1.10 (0.69, 1.75)	1.05 (0.63, 1.73)	1.42 (0.87, 2.31)
Obese	1.49 (0.58, 3.83)	1.48 (0.54, 4.02)	2.31 (0.90, 5.91)
* OR*_*trend*_	1.13 (0.79, 1.61)	1.13 (0.77, 1.66)	1.46 (1.02, 2.10)
* P trend*	0.49	0.53	0.04
* Family history*			
Absent	1.00 (reference)	1.00 (reference)	1.00 (reference)
Present	1.63 (0.73, 3.64)	0.52 (0.17, 1.59)	1.10 (0.43, 2.77)
* P value*	0.97	0.25	0.84

OR and 95% CI were from polytomous logistic regression models (Q1 of NPI was the base (comparison) category) with mutual adjustments for parity, body mass index (BMI), age at menarche, and family history. Models were further adjusted for diagnosis year and breast cancer subtype

### Associations between breast cancer risk factors and levels of immunohistochemical 4 (IHC4) score

Of the risk factors assessed, only BMI demonstrated significant associations with IHC4 score. In contrast to the positive associations that we found between elevated BMI and high NPI, elevated BMI was inversely associated with the IHC4 score. In comparison to normal weight women, overweight (OR (95% CI) = 0.82 (0.66–1.02)) and obese (OR (95% CI) = 0.52 (0.36–0.76)) women were less likely to have tumors with high IHC4 score (Table 4). In stratified analysis by age, we found the inverse association between increasing BMI and the IHC4 to be statistically significantly stronger among older than younger women (P value for heterogeneity [P-het] = 0.006) but estimates were in the same direction (Supplementary Table 5). We did not find evidence for associations between age at menarche, parity, and breastfeeding with the IHC4. Overall, the results were similar in analysis accounting for missing values on covariates (Supplementary Table 6).

**Table 4:** Odds ratios (ORs) and 95% confidence intervals (CIs) for the associations between breast cancer risk factors and levels of the immunohistochemical 4 (IHC4) score among Chinese breast cancer patients with complete data (*n* = 3475)

Characteristic	IHC4 score						
	Q1 (reference)	Q2		Q3		Q4	
	N	N	OR(95% CI)	N	OR(95% CI)	N	OR(95% CI)
*Age, years*							
<35	28	54	1.00 (reference)	50	1.00 (reference)	52	1.00 (reference)
35–45	245	218	0.49 (0.29, 0.82)	175	0.44 (0.26, 0.74)	199	0.51 (0.30, 0.86)
45–55	310	255	0.48 (0.29, 0.80)	284	0.61 (0.37, 1.03)	336	0.77 (0.46, 1.30)
>55	317	337	0.64 (0.38, 1.06)	323	0.68 (0.41, 1.15)	292	0.67 (0.40, 1.14)
OR_trend_			1.04 (0.93, 1.16)		1.10 (0.98, 1.24)		1.05 (0.93, 1.18)
* P trend*			0.54		0.09		0.42
*Age at Menarche, years*							
≤12	97	116	1.00 (reference)	88	1.00 (reference)	90	1.00 (reference)
13	179	141	0.65 (0.45, 0.93)	171	1.02 (0.70, 1.48)	177	1.07 (0.73, 1.56)
14	198	204	0.86 (0.61, 1.21)	200	1.09 (0.76, 1.56)	202	1.13 (0.77, 1.63)
≥15	426	403	0.77 (0.56, 1.06)	373	0.88 (0.63, 1.24)	410	1.01 (0.71, 1.42)
OR_trend_			0.96 (0.87, 1.05)		0.94 (0.85, 1.04)		0.99 (0.90, 1.10)
* P trend*			0.39		0.21		0.91
*Parity*							
Nulliparous	47	27	1.00 (reference)	29	1.00 (reference)	39	1.00 (reference)
1	460	438	1.94 (1.16, 3.24)	385	1.55 (0.93, 2.57)	427	1.25 (0.77, 2.02)
2	247	225	1.70 (1.00, 2.90)	240	1.60 (0.94, 2.72)	238	1.11 (0.66, 1.85)
≥3	146	174	2.01 (1.15, 3.53)	178	1.79 (1.03, 3.13)	175	1.19 (0.69, 2.04)
OR_trend_			1.06 (0.93, 1.21)		1.11 (0.97, 1.26)		0.98 (0.85, 1.12)
* P trend*			0.37		0.13		0.76
*Breastfeeding*							
Never	108	87	1.00 (reference)	82	1.00 (reference)	92	1.00 (reference)
Ever	686	660	1.20 (0.88, 1.65)	640	1.26 (0.91, 1.74)	679	1.17 (0.84, 1.62)
* P value*			0.25		0.16		0.36
*BMI*							
Underweight	9	17	2.03 (0.88, 4.69)	18	2.37 (1.02, 5.47)	14	1.67 (0.68, 4.08)
Normal	474	458	1.00 (reference)	441	1.00 (reference)	475	1.00 (reference)
Overweight	328	308	0.88 (0.71, 1.08)	306	0.85 (0.68, 1.05)	327	0.82 (0.66, 1.02)
Obese	89	81	0.79 (0.56, 1.10)	67	0.61 (0.43, 0.87)	63	0.52 (0.36, 0.76)
OR_trend_			0.89 (0.77, 1.03)		0.81 (0.70, 0.95)		0.77 (0.66, 0.91)
* P trend*			0.12		0.009		0.001
*Family history*							
Absent	820	799	1.00 (reference)	767	1.00 (reference)	822	1.00 (reference)
Present	80	65	0.88 (0.62, 1.25)	65	1.00 (0.70, 1.43)	57	0.87 (0.59, 1.27)
* P value*			0.47		0.99		0.46

Odds ratios (ORs) and 95% confidence intervals (CIs) were obtained using polytomous logistic regression models that were mutually adjusted for age, age at menarche, parity, body mass index (BMI) and family history of breast cancer in a first degree relative. Parity was adjusted in the main model (presented in table) and this was substituted for breastfeeding in a separate model. All models were further adjusted for year at diagnosis and the NPI score in complete case analysis. Overall, a total of 3475 subjects with complete information on IHC4, NPI, and breast cancer risk factors were included in the analysis presented in this table. In sensitivity analyses including missing value indicators for breast cancer risk factors and NPI (*n* = 7685; Supplementary Table 6), the results were similar to those presented here

### Age at onset, parity, BMI and FHBC in relation to NPI and IHC4 score among Polish breast cancer patients

To check whether the associations we found between age at onset, parity, BMI, and family history with clinical prognostic markers were seen in other populations, we used data from a subset of 972 Polish breast cancer patients. Similar to the findings among Chinese patients, increasing age and a positive FHBC in a first degree relative were associated with lower NPI values even though the estimates did not attain statistical significance. In addition, overweight (OR (95% CI) = 1.34 (0.82–2.19) and obese (OR (95% CI) = 2.27 (1.32–3.89) women were more likely to have high NPI when compared to normal weight women (Table 5), which is similar to what we found among the Chinese women. None of the factors evaluated was statistically significantly associated with the IHC4 score among Polish women overall. However, consistent with the findings among Chinese women, the inverse association between elevated BMI and levels of IHC4 score were stronger among older than younger women (P-het = 0.04; Supplementary Table 7).

**Table 5:** Odds ratios (ORs) and 95% confidence intervals (CIs) for the associations between age, parity, body mass index (BMI), family history, and levels of the Nottingham prognostic index (NPI) and immunohistochemical 4 (IHC4) score among Polish breast cancer patients (*n* = 972)

Characteristic	NPI			IHC4		
	Q2	Q3	Q4	Q2	Q3	Q4
	OR(95% CI)	OR(95% CI)	OR(95% CI)	OR(95% CI)	OR(95% CI)	OR(95% CI)
*Age, years*						
<45	1.00 (reference)	1.00 (reference)	1.00 (reference)	1.00 (reference)	1.00 (reference)	1.00 (reference)
45–55	0.74 (0.39, 1.41)	0.77 (0.41, 1.47)	0.72 (0.37, 1.37)	1.16 (0.57, 2.35)	0.69 (0.36, 1.34)	0.63 (0.32, 1.24)
55–65	0.59 (0.30, 1.14)	0.60 (0.31, 1.18)	0.45 (0.22, 0.89)	1.64 (0.78, 3.44)	0.90 (0.45, 1.83)	0.70 (0.34, 1.45)
>65	1.08 (0.54, 2.17)	0.89 (0.43, 1.82)	0.77 (0.37, 1.59)	1.17 (0.55, 2.51)	0.58 (0.28, 1.20)	0.50 (0.34, 1.06)
OR_trend_	1.05 (0.85, 1.28)	0.96 (0.78, 1.19)	0.91 (0.74, 1.14)	1.04 (0.84, 1.29)	0.89 (0.72, 1.11)	0.84 (0.67, 1.06)
* P trend*	0.67	0.74	0.43	0.68	0.30	0.14
*Parity*						
Nulliparous	1.00 (reference)	1.00 (reference)	1.00 (reference)	1.00 (reference)	1.00 (reference)	1.00 (reference)
1	1.05 (0.58, 1.91)	1.50 (0.84, 2.69)	1.34 (0.59, 3.03)	0.87 (0.49, 1.45)	1.06 (0.58, 1.55)	1.18 (0.63, 2.22)
2	0.79 (0.45, 1.38)	0.80 (0.46, 1.41)	0.86 (0.38, 1.93)	0.69 (0.39, 1.22)	1.04 (0.58, 1.87)	1.07 (0.58, 1.99)
≥3	0.72 (0.41, 1.29)	0.82 (0.46, 1.45)	1.05 (0.47, 2.36)	1.20 (0.55, 2.63)	0.86 (0.36, 2.05)	1.33 (0.56, 3.15)
OR_trend_	1.18 (0.95, 1.47)	0.95 (0.76, 1.18)	1.01 (0.80, 1.27)	0.95 (0.76, 1.20)	0.98 (0.78, 1.23)	1.05 (0.82, 1.33)
* P value*	0.14	0.63	0.96	0.69	0.87	0.71
*BMI*						
Underweight	1.44 (0.30, 6.95)	1.85 (0.39, 8.78)	0.42 (0.04, 4.43)	1.77 (0.30, 10.39)	1.67 (0.26, 10.60)	2.00 (0.32, 12.51)
Normal	1.00 (reference)	1.00 (reference)	1.00 (reference)	1.00 (reference)	1.00 (reference)	1.00 (reference)
Overweight	1.00 (0.63, 1.58)	1.27 (0.80, 2.03)	1.34 (0.82, 2.19)	1.08 (0.67, 1.73)	0.95 (0.58, 1.54)	0.82 (0.49, 1.36)
Obese	1.33 (0.80, 2.23)	1.58 (0.93, 2.69)	2.27 (1.32, 3.89)	0.81 (0.47, 1.38)	1.23 (0.72, 2.08)	0.81 (0.47, 1.43)
OR_trend_	1.13 (0.88, 1.46)	1.24 (0.96, 1.61)	1.48 (1.13, 1.93)	0.92 (0.70, 1.19)	1.11 (0.85, 1.45)	0.89 (0.67, 1.06)
* P trend*	0.34	0.10	0.004	0.51	0.43	0.44
*Family history*						
Absent	1.00 (reference)	1.00 (reference)	1.00 (reference)	1.00 (reference)	1.00 (reference)	1.00 (reference)
Present	1.00 (0.53, 1.90)	0.89 (0.46, 1.72)	0.65 (0.32, 1.32)	0.86 (0.44, 1.69)	1.14 (0.60, 2.18)	1.05 (0.52, 2.11)
* P value*	0.99	0.73	0.24	0.67	0.68	0.89

OR and 95% CI were from polytomous logistic regression models (Q1 was the base (comparison) category) with mutual adjustments for age, parity, body mass index (BMI), age at menarche (not shown), and family history of breast cancer in a first degree relative. The NPI model was further adjusted for breast cancer subtype while the IHC4 model was adjusted for NPI

### Associations between parity, BMI, and family history with individual clinicopathologic characteristics

In analysis to determine the contributions of individual clinicopathologic factors to predictive models for BMI, parity, and family history in relation to clinical prognostic markers, we modeled each of these risk factors as an outcome variable and clinicopathological and IHC factors as explanatory variables. In stepwise analyses, we removed each clinicopathologic factor from the full model and assessed the change in likelihood ration Chi-square (ΔLRχ^2^), which was compared with the nested model using LR tests. In general, age was most relevant for the estimation of models for BMI, parity, and FHBC. This was followed by tumor size, ER, HER2 and grade for obesity; node status, grade, tumor size and ER for parity; and node status, HER2, and grade for FHBC (Table 6).

**Table 6:** Change in likelihood ratio Chi-square (ΔLRχ^2^) and corresponding p values testing the contributions of individual clinicopathological characteristics to predictive models for body mass index (BMI), parity, and family history among Chinese breast cancer patients

Characteristic	Overweight		Obese		Parous		Positive FHBC	
	ΔLRχ^2^	*P value*	ΔLRχ^2^	*P value*	ΔLRχ^2^	*P value*	ΔLRχ^2^	*P value*
Age, per year	75.4	<0.001	40.0	<0.001	71.5	<0.001	4.7	0.03
Tumor size, per cm	4.0	0.04	10.7	0.001	2.3	0.13	0.3	0.29
Grade	0.4	0.81	5.0	0.08	3.4	0.18	1.5	0.47
Node	7.6	0.02	1.0	0.60	3.6	0.17	8.5	0.01
ER	4.0	0.04	9.9	0.002	2.1	0.15	0.0	0.97
PR	3.3	0.07	2.6	0.11	0.8	0.37	0.1	0.80
HER2	0.4	0.51	6.5	0.01	0.1	0.73	1.9	0.17

All risk factors were mutually adjusted for one another in unconditional logistic regression models separately for overweight (vs normal weight), obesity (vs normal weight), parous (vs nulliparous), family history of breast cancer (vs no family history) as dependent variables and the clinicopathological characteristics as predictor variables. We determined the change in likelihood ratio Chi-square by removing each factor from the full predictive model and comparing the nested model with the full model using a likelihood ratio test

## Discussion

In this large-scale analysis of over 8,000 Chinese breast cancer patients, we assessed relationships between breast cancer risk factors and clinically important combinatorial prognostic biomarkers in breast cancer. Specifically, we used routinely available clinicopathological (tumor size, histologic grade, or nodal involvement) and IHC (ER, PR, HER2, and KI67) parameters to compute the NPI and IHC4 score and investigated relationships with breast cancer risk factors. We found younger age, higher parity, being overweight or obese, and not having a FHBC to be associated with more clinically advanced breast cancer, represented by high NPI. Unlike what we observed in relation to the NPI, however, being overweight or obese was associated with lower IHC4 score, indicating less aggressive breast cancer phenotype. Nevertheless, accounting for HR expression, women with elevated BMI were more likely to have clinically advanced HR + and HR− breast cancers. Taken together, these results suggest that breast cancer risk factors may influence the biology and clinical presentation of breast tumors, with implications for improved surveillance and the development of prognostic tools incorporating risk factors.

In addition to tumor-related factors, the age at breast cancer diagnosis has long been recognized to be associated with worse clinical outcomes [21–25]. Our age-related findings are in keeping with those from previous studies demonstrating associations between younger age at onset and more aggressive tumor characteristics even among HR + tumors [21] and suggests that previously reported associations may partly be mediated by tumor characteristics, particularly those contained in the NPI, i.e. size, grade, and lymph nodal involvement. Nevertheless, further studies incorporating mediation analysis will be required to conclusively determine whether previously reported associations between age at onset and recurrence or survival following breast cancer are mediated by the influence of age on specific tumor characteristics.

Our findings of associations between increasing parity and clinically advanced breast cancer is in line with previously reported associations between parity and aggressive breast cancer phenotypes [26–28]. Given that IHC markers and other tumor characteristics are highly correlated, it is unclear if previously reported associations between parity and HR– tumors are driven entirely by HR expression or by other clinicopathological characteristics. By accounting for many of these markers in the current analysis, we demonstrated that tumor characteristics contained in the NPI may partly explain previously observed associations between parity and HR− breast cancer. The mechanism through which parity predisposes to aggressive phenotypes of breast cancer is largely unknown but it is thought to be related, at least in part, to changes in the breast microenvironment that can result from aberrant post-partum lobular involution [29, 30]. Previous reports were in support of the attenuating effects of prolonged breastfeeding on the association between parity and TNBC, an aggressive subtype of breast cancer [27, 31, 32]. In contrast to these reports, however, we did not find evidence to support associations between breastfeeding and any of the prognostic parameters.

Results from epidemiological studies indicate that elevated BMI more strongly predisposes to HR− than HR + breast cancers among premenopausal women, with the converse predominating among postmenopausal women [33, 34]. Given the superior survival indices for HR + over HR− breast cancer, some researchers have suggested that elevated BMI more strongly predisposes to less aggressive tumor subtypes, particularly in postmenopausal women [35]. Perhaps paradoxically, results from several other studies, including a large-scale pooling analysis, have supported higher frequencies of aggressive tumor characteristics, particularly histologic grade, among overweight and/or obese than normal weight women [9, 10, 36]. Our findings that elevated BMI was associated with low IHC4 on the one hand, and high NPI on the other hand, might explain previous reports since low IHC4 corresponds to high HR expression whereas high NPI can connote larger tumor size, higher histologic grade and/or positive lymph nodal status, all markers of poor prognosis. Notably, the associations between BMI and NPI were irrespective of HR expression, suggesting that overweight and/or obese women were more likely to develop aggressive tumor subtypes regardless of HR expression status. Our observation that several tumor characteristics were statistically significantly predictive of overweight and obese BMI following LR tests indicates that the somewhat paradoxical relationship between BMI and tumor aggressiveness might be due to varying roles of estrogen metabolism, adiponectin, insulin-like growth factors, chronic inflammation, and/or delayed detection in obesity-related breast carcinogenesis [29, 37–39].

The literature on the relationship between FHBC and clinical outcomes in breast cancer is not consistent [40–44]. In the current study, we observed having a positive FHBC to be associated with lower NPI values, suggesting better prognosis. In analysis assessing the contributions of the individual tumor characteristics to a predictive model for FHBC, we found lymph nodal status to provide more predictive information than other tumor characteristics. This might suggest that women with a positive FHBC were less likely to have clinically advanced disease than those without a FHBC. A possible explanation for this finding may be that women with a positive FHBC may be more likely to seek and/or undergo screening or other forms of surveillance which can lead to the detection of early stage tumors than those without a FHBC. This may be especially true in countries where large-scale, organized, breast cancer screening is not available and where selective screening is offered to high risk women (partly defined by having a positive FHBC). Accordingly, further studies with detailed screening histories will be required to conclusively determine relationships between FHBC, prognostic biomarkers, and clinical outcomes in breast cancer.

We assessed the external generalizability of findings from the Chinese population by investigating the key results in an independent population of Polish breast cancer patients. With the exception of parity, the associations between risk factors and NPI were in the same direction in the Polish and Chinese populations. In particular, the associations between BMI and NPI were significant in both populations. Unlike Chinese patients, for whom increasing parity was statistically significantly associated with higher NPI, parity was not associated with higher NPI among the Polish patients. Given the documented associations between pregnancy-associated breast cancer, which could occur up to 10 years after pregnancy, and aggressive phenotypes of breast cancer [45], it is possible that differences in results between the study populations may reflect differences in time since last childbirth. We were unable to specifically evaluate this question due to lack of information on time since last birth and breast cancer diagnosis in both study populations. Nonetheless, Polish participants were older and more likely to be postmenopausal than the Chinese participants which might suggest a longer time since last birth for the Polish patients. Future studies will be warranted to investigate the impact of time since last birth on clinically relevant prognostic biomarkers in breast cancer.

An important consideration for the development of targeted prevention strategies in breast cancer is the identification of women at high risk of developing the most aggressive subtypes of breast cancer, for whom interventions to prevent invasive disease can be instituted. Based on our findings, higher parity as well as being overweight or obese were associated with poor prognostic indices, suggesting a potential beneficial effect for improved surveillance among such women. Relatedly, these findings may be suggestive of the importance of including breast cancer risk factors as part of breast cancer prognostic models. To date, only PREDICT has included breast cancer risk factors, i.e. age at diagnosis and mode of detection, in its calculations and has been shown to outperform the IHC4 score, IHC subtyping, and C-score in terms of prognostic power [46]. It is probable that future models incorporating BMI may provide further survival discrimination in breast cancer patients beyond what is contained in existing models.

The assembly of several clinicopathological and epidemiological factors for >8000 patients is an important strength of this study. In terms of limitations, however, this population is largely unscreened, as such, some of the reported associations may be subject to detection bias. For example, smaller tumors may be less likely to be palpated in obese than normal weight women. Accordingly, overweight and/or obese women may be more likely to have larger tumors on the basis of detection alone. Nevertheless, other tumor characteristics were independently associated with higher BMI in this study, suggesting some biological underpinning for our findings. Moreover, detection bias is itself a clinically important problem that needs to be addressed. Also, we did not have clinical outcome data such as recurrence or survival for these patients, which precluded our ability to perform mediation analyses or to directly examine associations between breast cancer risk factors and clinical outcomes. Nonetheless, NPI and IHC4 score strongly predict clinical outcomes in breast cancer. Together, they reflect the spectrum of clinicopathological characteristics that are used in routine clinical practice to inform breast cancer management.

## Conclusions

In conclusion, our results support associations between breast cancer risk factors and two clinically useful prognostic factors in breast cancer, i.e. NPI and IHC4. Specifically, we found younger age at onset, higher parity, being overweight/obese and absent FHBC to be associated with poor prognostic indices, indicated by higher NPI score, irrespective of HR expression. Although elevated BMI was associated with low IHC4 score, connoting clinically less aggressive HR + tumors, NPI-related findings were supportive of poor prognosis among overweight/obese patients irrespective of tumor HR status. These findings highlight important relationships between breast cancer risk factors and clinically relevant prognostic factors, with potential implications for the development of surveillance, prognostication, and counseling strategies that take host factors into account.

## Supplementary Information

Below is the link to the electronic supplementary material.

Supplementary file1 (DOCX 72 kb)

## Data Availability

All the datasets used and/or analyzed during the current study are available from the corresponding author on reasonable request.

## Abbreviations

BMI: Body mass index
CHCAMS: Cancer Hospital, Chinese Academy of Medical Sciences
ER: Estrogen receptor
FHBC: Family history of breast cancer
HR +: Hormone receptor-positive
HR−: Hormone receptor-negative
IHC: Immunohistochemical/immunohistochemistry
OR: Odds ratio
PBCS: Polish Breast Cancer Study
PR: Progesterone receptor
NPI: Nottingham Prognostic Index
