# Supplementary material for: Impact of breast cancer risk factors on clinically relevant prognostic biomarkers for primary breast cancer
Source: Breast Cancer Res Treat. 2021 Jun 29;189(2):483–95. doi: 10.1007/s10549-021-06294-5 (PMC8357643; doi:10.1007/s10549-021-06294-5)
Supplement: Supplementary file 1 — Supplementary file1 (DOCX 72 kb) [file 10549_2021_6294_MOESM1_ESM.docx]

**Supplementary Table 1:** Distributions of tumor clinicopathological and breast cancer risk factors overall and by tumor hormone receptor expression status among Polish breast cancer patients

|  |  | **Overall (n=972)** | | |  | **HR+ (n=713)** | | |  | **HR- (n=253)** | | |  |
| --- | --- | --- | --- | --- | --- | --- | --- | --- | --- | --- | --- | --- | --- |
| **Characteristic** |  | **Freq** |  | **%** |  | **Freq** |  | **%** |  | **Freq** |  | **%** | ***P value*** |
| Age, years |  |  |  |  |  |  |  |  |  |  |  |  |  |
| <35 |  | 11 |  | 1.1 |  | 6 |  | 0.8 |  | 5 |  | 2.0 |  |
| 35-45 |  | 138 |  | 14.2 |  | 94 |  | 13.2 |  | 43 |  | 17.0 |  |
| 45-55 |  | 367 |  | 37.7 |  | 271 |  | 38.0 |  | 93 |  | 36.8 |  |
| >55 |  | 456 |  | 47.0 |  | 342 |  | 48.0 |  | 112 |  | 44.2 | 0.20 |
| Histologic grade |  |  |  |  |  |  |  |  |  |  |  |  |  |
| Low |  | 181 |  | 18.7 |  | 167 |  | 23.4 |  | 13 |  | 5.1 |  |
| Intermediate |  | 546 |  | 56.3 |  | 454 |  | 63.7 |  | 90 |  | 35.6 |  |
| High |  | 242 |  | 25.0 |  | 92 |  | 12.9 |  | 150 |  | 59.3 | <0.001 |
| Size |  |  |  |  |  |  |  |  |  |  |  |  |  |
| ≤2cm |  | 482 |  | 49.9 |  | 394 |  | 55.3 |  | 86 |  | 34.3 |  |
| >2cm |  | 484 |  | 50.1 |  | 318 |  | 44.7 |  | 165 |  | 65.7 | <0.001 |
| Nodal involvement |  |  |  |  |  |  |  |  |  |  |  |  |  |
| None |  | 531 |  | 55.7 |  | 389 |  | 55.6 |  | 140 |  | 55.8 |  |
| 1 |  | 148 |  | 15.5 |  | 112 |  | 16.0 |  | 36 |  | 14.3 |  |
| 2 |  | 69 |  | 7.2 |  | 56 |  | 8.0 |  | 13 |  | 5.2 |  |
| ≥3 |  | 206 |  | 21.6 |  | 143 |  | 20.4 |  | 62 |  | 24.7 | 0.27 |
| ER |  |  |  |  |  |  |  |  |  |  |  |  |  |
| Negative |  | 301 |  | 31.6 |  |  |  |  |  |  |  |  |  |
| Positive |  | 661 |  | 68.4 |  |  |  |  |  |  |  |  |  |
| PR |  |  |  |  |  |  |  |  |  |  |  |  |  |
| Negative |  | 473 |  | 49.0 |  |  |  |  |  |  |  |  |  |
| Positive |  | 493 |  | 51.0 |  |  |  |  |  |  |  |  |  |
| HER2 |  |  |  |  |  |  |  |  |  |  |  |  |  |
| Negative |  | 783 |  | 83.3 |  | 611 |  | 88.0 |  | 171 |  | 70.1 |  |
| Positive |  | 157 |  | 16.7 |  | 83 |  | 12.0 |  | 73 |  | 29.9 | <0.001 |
| KI67 |  |  |  |  |  |  |  |  |  |  |  |  |  |
| low |  | 549 |  | 56.5 |  | 458 |  | 64.2 |  | 87 |  | 34.4 |  |
| High |  | 423 |  | 43.5 |  | 255 |  | 35.8 |  | 166 |  | 65.6 | <0.001 |
| Age at Menarche, years |  |  |  |  |  |  |  |  |  |  |  |  |  |
| ≤12 |  | 275 |  | 28.7 |  | 194 |  | 27.5 |  | 80 |  | 32.1 |  |
| 13 |  | 217 |  | 22.7 |  | 164 |  | 23.3 |  | 53 |  | 21.3 |  |
| 14 |  | 256 |  | 26.8 |  | 185 |  | 26.2 |  | 70 |  | 28.1 |  |
| ≥15 |  | 209 |  | 21.8 |  | 162 |  | 23.0 |  | 46 |  | 18.5 | 0.30 |
| Parity |  |  |  |  |  |  |  |  |  |  |  |  |  |
| Nulliparous |  | 154 |  | 16.0 |  | 119 |  | 16.8 |  | 35 |  | 13.9 |  |
| 1 |  | 329 |  | 34.1 |  | 240 |  | 33.9 |  | 87 |  | 34.5 |  |
| 2 |  | 394 |  | 40.9 |  | 286 |  | 40.3 |  | 107 |  | 42.5 |  |
| ≥3 |  | 87 |  | 9.0 |  | 64 |  | 9.0 |  | 23 |  | 9.1 | 0.75 |
| Body mass index (BMI) |  |  |  |  |  |  |  |  |  |  |  |  |  |
| Underweight |  | 13 |  | 1.3 |  | 9 |  | 1.3 |  | 4 |  | 1.6 |  |
| Normal |  | 295 |  | 30.7 |  | 210 |  | 29.7 |  | 84 |  | 33.5 |  |
| Overweight |  | 351 |  | 36.5 |  | 267 |  | 37.7 |  | 84 |  | 33.5 |  |
| Obese |  | 303 |  | 31.5 |  | 222 |  | 31.3 |  | 79 |  | 31.4 | 0.59 |
| Family history |  |  |  |  |  |  |  |  |  |  |  |  |  |
| Absent |  | 871 |  | 90.5 |  | 664 |  | 91.2 |  | 223 |  | 88.5 |  |
| Present |  | 92 |  | 9.5 |  | 62 |  | 8.8 |  | 29 |  | 11.5 | 0.20 |

**P values* were obtained from Chi-squared tests for categorical variables.

**Supplementary Table 2:** Odds ratios (ORs) and 95% confidence intervals (CIs) for the associations between patient-related lifestyle and reproductive factors and levels of PREDICT among Chinese breast cancer patients

|  |  | **PREDICT** | | | | | | | | | |
| --- | --- | --- | --- | --- | --- | --- | --- | --- | --- | --- | --- |
|  |  | **Q1 (reference)** |  | **Q2** | |  | **Q3** | |  | **Q4** | |
| **Characteristic** |  | **N** |  | **N** | **OR (95% CI)** |  | **N** | **OR (95% CI)** |  | **N** | **OR (95% CI)** |
| **Age, years** |  |  |  |  |  |  |  |  |  |  |  |
| <35 |  | 24 |  | 38 | 1.00 (reference) |  | 65 | 1.00 (reference) |  | 121 | 1.00 (reference) |
| 35-45 |  | 266 |  | 250 | 0.55 (0.30, 1.02) |  | 276 | 0.32 (0.18, 0.58) |  | 224 | 0.13 (0.07, 0.23) |
| 45-55 |  | 430 |  | 352 | 0.43 (0.24, 0.80) |  | 356 | 0.20 (0.11, 0.36) |  | 347 | 0.10 (0.06, 0.17) |
| >55 |  | 362 |  | 443 | 0.64 (0.35, 1.19) |  | 388 | 0.27 (0.15, 0.48) |  | 389 | 0.14 (0.08, 0.24) |
| OR_trend_ |  |  |  |  | 1.01 (0.91, 1.14) |  |  | 0.83 (0.74, 0.94) |  |  | 0.78 (0.70, 0.88) |
| *P trend* |  |  |  |  | 0.72 |  |  | 0.002 |  |  | <0.001 |
| **Age at Menarche, years** |  |  |  |  |  |  |  |  |  |  |  |
| ≤12 |  | 114 |  | 102 | 1.00 (reference) |  | 109 | 1.00 (reference) |  | 98 | 1.00 (reference) |
| 13 |  | 191 |  | 192 | 1.03 (0.73, 1.47) |  | 174 | 0.82 (0.57, 1.18) |  | 164 | 0.87 (0.60, 1.28) |
| 14 |  | 224 |  | 230 | 1.05 (0.74, 1.47) |  | 231 | 0.92 (0.65, 1.31) |  | 206 | 0.94 (0.65, 1.36) |
| ≥15 |  | 434 |  | 440 | 1.00 (0.73, 1.38) |  | 442 | 0.91 (0.66, 1.27) |  | 445 | 1.02 (0.73, 1.44) |
| OR_trend_ |  |  |  |  | 0.99 (0.90, 1.08) |  |  | 1.00 (0.91, 1.10) |  |  | 1.03 (0.94, 1.14) |
| *P* *trend* |  |  |  |  | 0.81 |  |  | 0.97 |  |  | 0.50 |
| **Parity** |  |  |  |  |  |  |  |  |  |  |  |
| Nulliparous |  | 40 |  | 41 | 1.00 (reference) |  | 36 | 1.00 (reference) |  | 44 | 1.00 (reference) |
| 1 |  | 543 |  | 505 | 0.86 (0.53, 1.40) |  | 447 | 1.02 (0.60, 1.72) |  | 436 | 0.94 (0.55, 1.59) |
| 2 |  | 239 |  | 273 | 0.90 (0.54, 1.50) |  | 291 | 1.39 (0.80, 2.41) |  | 266 | 1.15 (0.66, 1.99) |
| ≥3 |  | 260 |  | 264 | 0.79 (0.46, 1.35) |  | 311 | 1.47 (0.82, 2.60) |  | 335 | 1.37 (0.77, 2.43) |
| OR_trend_ |  |  |  |  | 0.97 (0.85, 1.11) |  |  | 1.23 (1.07, 1.40) |  |  | 1.20 (1.05, 1.38) |
| *P* *trend* |  |  |  |  | 0.67 |  |  | 0.002 |  |  | 0.007 |
| **Breastfeeding** |  |  |  |  |  |  |  |  |  |  |  |
| Never |  | 115 |  | 111 | 1.00 (reference) |  | 86 | 1.00 (reference) |  | 104 | 1.00 (reference) |
| Ever |  | 750 |  | 722 | 0.90 (0.67, 1.22) |  | 733 | 1.29 (0.93, 1.81) |  | 674 | 0.99 (0.71, 1.38) |
| *P value* |  |  |  |  | 0.50 |  |  | 0.13 |  |  | 0.95 |
| **BMI** |  |  |  |  |  |  |  |  |  |  |  |
| Underweight |  | 23 |  | 15 | 0.73 (0.36, 1.51) |  | 15 | 0.78 (0.37, 1.65) |  | 21 | 1.18 (0.59, 2.35) |
| Normal |  | 583 |  | 540 | 1.00 (reference) |  | 485 | 1.00 (reference) |  | 482 | 1.00 (reference) |
| Overweight |  | 315 |  | 355 | 1.25 (1.02, 1.54) |  | 387 | 1.63 (1.32, 2.01) |  | 372 | 1.57 (1.26, 1.94) |
| Obese |  | 54 |  | 87 | 1.72 (1.17, 2.50) |  | 98 | 2.35 (1.60, 3.43) |  | 90 | 2.07 (1.40, 3.07) |
| OR_trend_ |  |  |  |  | 1.29 (1.11, 1.51) |  |  | 1.57 (1.34, 1.84) |  |  | 1.50 (1.28, 1.76) |
| *P trend* |  |  |  |  | 0.001 |  |  | <0.001 |  |  | <0.001 |
| **Family history** |  |  |  |  |  |  |  |  |  |  |  |
| Absent |  | 872 |  | 929 | 1.00 (reference) |  | 906 | 1.00 (reference) |  | 913 | 1.00 (reference) |
| Present |  | 106 |  | 60 | 0.56 (0.40, 0.80) |  | 78 | 0.79 (0.56, 1.11) |  | 50 | 0.48 (0.32, 0.71) |
| *P value* |  |  |  |  | 0.001 |  |  | 0.18 |  |  | <0.001 |

ORs and 95% CIs were from polytomous logistic regression models (Q1 was the base (comparison) category) with mutual adjustments for age, parity, age at menarche, BMI and family history. Parity was adjusted in the main model (presented in table) and this was substituted for breastfeeding in a separate model. All models were further adjusted for year of diagnosis and breast cancer subtype.

**Supplementary Table 3:** Odds ratios (ORs) and 95% confidence intervals (CIs) for the associations between parity, BMI, and family history and levels of the Nottingham prognostic index (NPI) stratified by age (≤50 vs >50 years) among Chinese breast cancer patients

|  |  | **NPI** | | | | |
| --- | --- | --- | --- | --- | --- | --- |
|  |  | **Q2 *vs* Q1** |  | **Q3 *vs* Q1** |  | **Q4 *vs* Q1** |
| **Characteristic** |  | **OR (95% CI)** |  | **OR (95% CI)** |  | **OR (95% CI)** |
| **Women ≤50 years** | |  |  |  |  |  |
| **Parity** |  |  |  |  |  |  |
| Nulliparous |  | 1.00 (reference) |  | 1.00 (reference) |  | 1.00 (reference) |
| 1 |  | 0.82 (0.46, 1.46) |  | 0.92 (0.50, 1.68) |  | 1.16 (0.60, 2.24) |
| 2 |  | 0.74 (0.39, 1.41) |  | 1.09 (0.56, 2.12) |  | 1.40 (0.68, 2.85) |
| ≥3 |  | 0.58 (0.28, 1.23) |  | 1.38 (0.66, 2.90) |  | 2.20 (1.02, 4.78) |
| *P trend* |  | 0.14 |  | 0.16 |  | 0.01 |
|  |  |  |  |  |  |  |
| **BMI** |  |  |  |  |  |  |
| Underweight |  | 0.58 (0.22, 1.52) |  | 1.23 (0.54, 2.80) |  | 1.02 (0.43, 2.44) |
| Normal |  | 1.00 (reference) |  | 1.00 (reference) |  | 1.00 (reference) |
| Overweight |  | 1.71 (1.25, 2.34) |  | 1.63 (1.18, 2.25) |  | 1.74 (1.25, 2.42) |
| Obese |  | 3.31 (1.64, 6.69) |  | 3.70 (1.83, 7.48) |  | 3.32 (1.61, 6.85) |
| P trend |  | <0.001 |  | <0.001 |  | <0.001 |
|  |  |  |  |  |  |  |
| **Family history** |  |  |  |  |  |  |
| Absent |  | 1.00 (reference) |  | 1.00 (reference) |  | 1.00 (reference) |
| Present |  | 0.57 (0.35, 0.93) |  | 0.75 (0.47, 1.22) |  | 0.48 (0.28, 0.82) |
| *P value* |  | 0.02 |  | 0.24 |  | 0.007 |
|  |  |  |  |  |  |  |
| **Women >50 years** | |  |  |  |  |  |
| **Parity** |  |  |  |  |  |  |
| Nulliparous |  | 1.00 (reference) |  | 1.00 (reference) |  | 1.00 (reference) |
| 1 |  | 0.82 (0.35, 1.88) |  | 1.06 (0.41, 2.73) |  | 1.34 (0.49, 3.69) |
| 2 |  | 0.85 (0.37, 1.98) |  | 1.40 (0.54, 3.64) |  | 1.70 (0.61, 4.69) |
| ≥3 |  | 0.72 (0.30, 1.70) |  | 1.21 (0.46, 3.19) |  | 1.52 (0.54, 4.27) |
| *P trend* |  | 0.56 |  | 0.29 |  | 0.27 |
|  |  |  |  |  |  |  |
| **BMI** |  |  |  |  |  |  |
| Underweight |  | 1.08 (0.37, 3.15) |  | 0.12 (0.01, 1.05) |  | 0.97 (0.30, 3.16) |
| Normal |  | 1.00 (reference) |  | 1.00 (reference) |  | 1.00 (reference) |
| Overweight |  | 1.31 (0.99, 1.72) |  | 1.33 (1.00, 1.78) |  | 1.49 (1.11, 2.00) |
| Obese |  | 1.47 (0.92, 2.34) |  | 1.77 (1.10, 2.85) |  | 1.75 (1.08, 2.83) |
| *P trend* |  | 0.03 |  | 0.006 |  | 0.003 |
|  |  |  |  |  |  |  |
| **Family history** |  |  |  |  |  |  |
| Absent |  | 1.00 (reference) |  | 1.00 (reference) |  | 1.00 (reference) |
| Present |  | 0.92 (0.57, 1.48) |  | 0.45 (0.24, 0.82) |  | 0.86 (0.51, 1.44) |
| *P value* |  | 0.73 |  | 0.009 |  | 0.55 |
|  |  |  |  |  |  |  |

Odds ratios and 95% confidence intervals were from polytomous logistic regression models adjusted for age, parity, age at menarche, family history of breast cancer in a first degree relative, year of diagnosis, and breast cancer subtype.

**Supplementary Table 4:** Odds ratios (ORs) and 95% confidence intervals (CIs) for the associations between breast cancer risk factors and levels of the Nottingham prognostic index (NPI) among Chinese breast cancer patients after accounting for missing values on covariates (n=4,196).

|  |  | **NPI** | | | | | | | | | |
| --- | --- | --- | --- | --- | --- | --- | --- | --- | --- | --- | --- |
|  |  | **Q1 (reference)** |  |  | **Q2** |  |  | **Q3** |  |  | **Q4** |
| **Characteristic** |  | **N** |  | **N** | **OR (95% CI)** |  | **N** | **OR (95% CI)** |  | **N** | **OR (95% CI)** |
| **Age, years** |  |  |  |  |  |  |  |  |  |  |  |
| <35 |  | 32 |  | 52 | 1.00 (reference) |  | 66 | 1.00 (reference) |  | 85 | 1.00 (reference) |
| 35-45 |  | 228 |  | 244 | 0.65 (0.39, 1.06) |  | 265 | 0.53 (0.33, 0.86) |  | 243 | 0.36 (0.22, 0.58) |
| 45-55 |  | 365 |  | 360 | 0.53 (0.33, 0.87) |  | 358 | 0.37 (0.23, 0.59) |  | 362 | 0.28 (0.17, 0.44) |
| >55 |  | 370 |  | 441 | 0.67 (041, 1.09) |  | 368 | 0.37 (0.23, 0.60) |  | 357 | 0.26 (0.16, 0.42) |
| OR_trend_ |  |  |  |  | 0.97 (0.88, 1.08) |  |  | 0.80 (0.72, 0.89) |  |  | 0.77 (0.69, 0.85) |
| *P trend* |  |  |  |  | 0.65 |  |  | <0.001 |  |  | <0.001 |
| **Age at Menarche, years** |  |  |  |  |  |  |  |  |  |  |  |
| ≤12 |  | 106 |  | 103 | 1.00 (reference) |  | 100 | 1.00 (reference) |  | 100 | 1.00 (reference) |
| 13 |  | 175 |  | 196 | 1.12 (0.79, 1.60) |  | 179 | 1.01 (0.70, 1.46) |  | 153 | 0.90 (0.62, 1.30) |
| 14 |  | 209 |  | 243 | 1.18 (0.84, 1.66) |  | 212 | 1.04 (0.73, 1.48) |  | 200 | 0.99 (0.70, 1.42) |
| ≥15 |  | 403 |  | 452 | 1.09 (0.79, 1.50) |  | 436 | 1.07 (0.77, 1.49) |  | 430 | 1.05 (0.76, 1.47) |
| OR_trend_ |  |  |  |  | 1.01 (0.92, 1.1) |  |  | 1.02 (0.92, 1.12) |  |  | 1.04 (0.94, 1.14) |
| *P* trend |  |  |  |  | 0.81 |  |  | 0.71 |  |  | 0.48 |
| **Parity** |  |  |  |  |  |  |  |  |  |  |  |
| Nulliparous |  | 37 |  | 48 | 1.00 (reference) |  | 38 | 1.00 (reference) |  | 30 | 1.00 (reference) |
| 1 |  | 493 |  | 528 | 0.82 (0.52, 1.32) |  | 448 | 0.94 (0.57, 1.56) |  | 424 | 1.18 (0.69, 2.02) |
| 2 |  | 224 |  | 267 | 0.77 (0.47, 1.26) |  | 277 | 1.12 (0.66, 1.90) |  | 267 | 1.43 (0.82, 2.49) |
| ≥3 |  | 241 |  | 254 | 0.63 (0.37, 1.06) |  | 294 | 1.02 (0.59, 1.76) |  | 326 | 1.37 (0.77, 2.44) |
| OR_trend_ |  |  |  |  | 0.88 (0.77, 0.99) |  |  | 1.05 (0.92, 1.19) |  |  | 1.10 (0.97, 1.25) |
| *P* trend |  |  |  |  | 0.04 |  |  | 0.47 |  |  | 0.14 |
| **Breastfeeding** |  |  |  |  |  |  |  |  |  |  |  |
| Never |  | 105 |  | 110 | 1.00 (reference) |  | 91 | 1.00 (reference) |  | 91 | 1.00 (reference) |
| Ever |  | 700 |  | 730 | 0.94 (0.70, 1.28) |  | 704 | 1.16 (0.84, 1.60) |  | 665 | 1.10 (0.79, 1.52) |
| *P value* |  |  |  |  | 0.71 |  |  | 0.36 |  |  | 0.56 |
| **BMI** |  |  |  |  |  |  |  |  |  |  |  |
| Underweight |  | 21 |  | 16 | 0.67 (0.34, 1.33) |  | 16 | 0.73 (0.36, 1.47) |  | 18 | 0.89 (0.45, 1.74) |
| Normal |  | 546 |  | 526 | 1.00 (reference) |  | 496 | 1.00 (reference) |  | 461 | 1.00 (reference) |
| Overweight |  | 287 |  | 384 | 1.43 (1.17, 1.75) |  | 357 | 1.44 (1.17, 1.78) |  | 364 | 1.56 (1.26, 1.93) |
| Obese |  | 52 |  | 88 | 1.87 (1.28, 2.73) |  | 98 | 2.33 (1.60, 3.40) |  | 86 | 2.09 (1.42, 3.08) |
| OR_trend_ |  |  |  |  | 1.40 (1.20, 1.63) |  |  | 1.50 (1.28, 1.75) |  |  | 1.50 (1.28, 1.75) |
| *P trend* |  |  |  |  | <0.001 |  |  | <0.001 |  |  | <0.001 |
| **Family history** |  |  |  |  |  |  |  |  |  |  |  |
| Absent |  | 813 |  | 940 | 1.00 (reference) |  | 905 | 1.00 (reference) |  | 865 | 1.00 (reference) |
| Present |  | 94 |  | 72 | 0.72 (0.51, 1.00) |  | 59 | 0.62 (0.43, 0.90) |  | 61 | 0.70 (0.49, 1.00) |
| *P value* |  |  |  |  | 0.05 |  |  | 0.01 |  |  | 0.05 |

ORs and 95% CIs were from polytomous logistic regression models (Q1 was the base (comparison) category) with mutual adjustments for age, parity, age at menarche, BMI, and family history. Parity was adjusted in the main model (presented in table) and this was substituted for breastfeeding in a separate model. All models were further adjusted for year of diagnosis and breast cancer subtype.

**Supplementary Table 5:** Odds ratios (ORs) and 95% confidence intervals (CIs) for the associations between body mass index (BMI) and levels of the immunohistochemical 4 (IHC4) score stratified by age (≤50 vs >50 years) among Chinese breast cancer patients

|  | **≤50 years** | | | |  | **>50 years** | | | |  |  |
| --- | --- | --- | --- | --- | --- | --- | --- | --- | --- | --- | --- |
|  | **Overweight** | **Obese** |  |  |  | **Overweight** | **Obese** |  |  |  |  |
|  | **OR (95% CI)** | **OR (95% CI)** | ***OR trend*** | ***P trend*** |  | **OR (95% CI)** | **OR (95% CI)** | **OR trend** | ***P trend*** |  | ***P het*** |
| **IHC4 score** |  |  |  |  |  |  |  |  |  |  |  |
| Q1 | 1.00 (reference) | 1.00 (reference) | 1.00 (reference) |  |  | 1.00 (reference) | 1.00 (reference) | 1.00 (reference) |  |  |  |
| Q2 | 0.81 (0.59, 1.11) | 0.76 (0.43, 1.33) | 0.84 (0.67, 1.06) | 0.15 |  | 0.91 (0.68, 1.22) | 0.81 (0.53, 1.25) | 0.90 (0.74, 1.11) | 0.33 |  |  |
| Q3 | 1.01 (0.74, 1.40) | 0.77 (0.43, 1.40) | 0.94 (0.74, 1.19) | 0.61 |  | 0.69 (0.52, 0.92) | 0.51 (0.32, 0.79) | 0.70 (0.57, 0.86) | 0.001 |  |  |
| Q4 | 0.92 (0.67, 1.27) | 0.71 (0.40, 1.27) | 0.87 (0.70, 1.12) | 0.31 |  | 0.68 (0.50, 0.92) | 0.42 (0.26, 0.68) | 0.65 (0.53, 0.81) | <0.001 |  | 0.006 |
|  |  |  |  |  |  |  |  |  |  |  |  |

Odds ratios and 95% confidence intervals were from polytomous logistic regression models adjusted for age, parity, age at menarche, family history of breast cancer in a first degree relative, year of diagnosis, and NPI.

**Supplementary Table 6:** Odds ratios (ORs) and 95% confidence intervals (CIs) for the associations between breast cancer risk factors and levels of the immunohistochemical 4 (IHC4) score among Chinese breast cancer patients after accounting for missing values on covariates (n=7,685).

|  |  | **IHC4 score** | | | | | | | | | |
| --- | --- | --- | --- | --- | --- | --- | --- | --- | --- | --- | --- |
|  |  | **Q1 (reference)** |  |  | **Q2** |  | **Q3** | |  | **Q4** | |
| **Characteristic** |  | **N** |  | **N** | **OR(95% CI)** |  | **N** | **OR(95% CI)** |  | **N** | **OR(95% CI)** |
| **Age, years** |  |  |  |  |  |  |  |  |  |  |  |
| <35 |  | 73 |  | 91 | 1.00 (reference) |  | 119 | 1.00 (reference) |  | 104 | 1.00 (reference) |
| 35-45 |  | 522 |  | 457 | 0.74 (0.53, 1.04) |  | 408 | 0.51 (0.36, 0.70) |  | 440 | 0.64 (0.45, 0.89) |
| 45-55 |  | 704 |  | 571 | 0.70 (0.50, 0.98) |  | 652 | 0.63 (0.45, 0.86) |  | 734 | 0.83 (0.60, 1.16) |
| >55 |  | 719 |  | 692 | 0.85 (0.60, 1.19) |  | 733 | 0.69 (0.50, 0.95) |  | 639 | 0.71 (0.51, 0.99) |
| OR_trend_ |  |  |  |  | 1.02 (0.94, 1.12) |  |  | 1.07 (0.98, 1.16) |  |  | 1.00 (0.92, 1.09) |
| *P trend* |  |  |  |  | 0.61 |  |  | 0.15 |  |  | 0.94 |
| **Age at Menarche, years** |  |  |  |  |  |  |  |  |  |  |  |
| ≤12 |  | 178 |  | 185 | 1.00 (reference) |  | 157 | 1.00 (reference) |  | 144 | 1.00 (reference) |
| 13 |  | 301 |  | 257 | 0.83 (0.63, 1.08) |  | 291 | 1.08 (0.82, 1.42) |  | 290 | 1.20 (0.90, 1.59) |
| 14 |  | 378 |  | 317 | 0.81 (0.63, 1.05) |  | 330 | 0.98 (0.75, 1.28) |  | 320 | 1.07 (0.81, 1.41) |
| ≥15 |  | 792 |  | 691 | 0.84 (0.66, 1.07) |  | 701 | 0.96 (0.75, 1.23) |  | 747 | 1.17 (0.90, 1.59) |
| OR_trend_ |  |  |  |  | 0.96 (0.90, 1.03) |  |  | 0.96 (0.90, 1.03) |  |  | 1.04 (0.96, 1.12) |
| *P trend* |  |  |  |  | 0.31 |  |  | 0.29 |  |  | 0.33 |
| **Parity** |  |  |  |  |  |  |  |  |  |  |  |
| Nulliparous |  | 86 |  | 56 | 1.00 (reference) |  | 60 | 1.00 (reference) |  | 63 | 1.00 (reference) |
| 1 |  | 976 |  | 877 | 1.53 (1.07, 2.18) |  | 861 | 1.43 (1.00, 2.04) |  | 880 | 1.35 (0.95, 1.92) |
| 2 |  | 469 |  | 413 | 1.42 (0.98, 2.07) |  | 432 | 1.42 (0.98, 2.05) |  | 430 | 1.28 (0.88, 1.85) |
| ≥3 |  | 487 |  | 465 | 1.47 (1.00, 2.18) |  | 559 | 1.65 (1.12, 2.43) |  | 544 | 1.35 (0.92, 1.99) |
| OR_trend_ |  |  |  |  | 1.03 (0.93, 1.14) |  |  | 1.05 (0.95, 1.16) |  |  | 0.98 (0.88, 1.08) |
| *P trend* |  |  |  |  | 0.51 |  |  | 0.33 |  |  | 0.63 |
| **Breastfeeding** |  |  |  |  |  |  |  |  |  |  |  |
| Never |  | 192 |  | 146 | 1.00 (reference) |  | 155 | 1.00 (reference) |  | 155 | 1.00 (reference) |
| Ever |  | 1299 |  | 1139 | 1.18 (0.93, 1.50) |  | 1165 | 1.14 (0.90, 1.45) |  | 1190 | 1.14 (0.90, 1.45) |
| *P value* |  |  |  |  | 0.17 |  |  | 0.27 |  |  | 0.28 |
| **BMI** |  |  |  |  |  |  |  |  |  |  |  |
| Underweight |  | 24 |  | 34 | 1.59 (0.93, 2.71) |  | 36 | 1.60 (0.93, 2.72) |  | 34 | 1.56 (0.90, 2.70) |
| Normal |  | 925 |  | 850 | 1.00 (reference) |  | 894 | 1.00 (reference) |  | 883 | 1.00 (reference) |
| Overweight |  | 659 |  | 545 | 0.85 (0.73, 0.99) |  | 563 | 0.81 (0.69, 0.94) |  | 571 | 0.82 (0.70, 0.95) |
| Obese |  | 160 |  | 139 | 0.86 (0.67, 1.11) |  | 132 | 0.73 (0.57, 0.95) |  | 122 | 0.68 (0.52, 0.88) |
| OR_trend_ |  |  |  |  | 0.90 (0.80, 1.01) |  |  | 0.83 (0.74, 0.93) |  |  | 0.83 (0.74, 0.93) |
| *P trend* |  |  |  |  | 0.06 |  |  | 0.002 |  |  | 0.002 |
| **Family history** |  |  |  |  |  |  |  |  |  |  |  |
| Absent |  | 1619 |  | 1451 | 1.00 (reference) |  | 1506 | 1.00 (reference) |  | 1498 | 1.00 (reference) |
| Present |  | 143 |  | 126 | 1.00 (0.78, 1.29) |  | 118 | 0.95 (0.73, 1.24) |  | 119 | 1.02 (0.78, 1.32) |
| *P value* |  |  |  |  | 0.97 |  |  | 0.72 |  |  | 0.90 |

ORs and 95% CIs were from polytomous logistic regression models (Q1 was the base (comparison) category) with mutual adjustments for age, parity, age at menarche, BMI, and family history. Parity was adjusted in the main model (presented in table) and this was substituted for breastfeeding in a separate model. All models were further adjusted for year of diagnosis and NPI.

**Supplementary Table 7:** Odds ratios (ORs) and 95% confidence intervals (CIs) for associations between body mass index (BMI) and levels of the Nottingham prognostic index (NPI) and immunohistochemical 4 (IHC4) score, overall and following stratification by age (≤50 years vs >50 years) among patients in the Polish Breast Cancer Study (PBCS).

|  | **Overall (n=972)** | | |  | **≤50 years (n=298)** | |  | **>50 years (n=674)** | |  |  |
| --- | --- | --- | --- | --- | --- | --- | --- | --- | --- | --- | --- |
|  | **Overweight** | **Obese** |  |  | **Overweight/Obese** |  |  | **Overweight/Obese** |  |  |  |
|  | **OR (95% CI)** | **OR (95% CI)** | ***P trend*** |  | **OR (95% CI)** | ***P value*** |  | **OR (95% CI)** | ***P value*** |  | ***P het*** |
| **NPI** |  |  |  |  |  |  |  |  |  |  |  |
| Q1 | 1.00 (reference) | 1.00 (reference) |  |  | 1.00 (reference) |  |  | 1.00 (reference) |  |  |  |
| Q2 | 1.00 (0.63, 1.58) | 1.33 (0.80, 2.23) | 0.34 |  | 1.38 (0.65, 2.95) | 0.39 |  | 1.12 (0.66, 1.91) | 0.68 |  |  |
| Q3 | 1.27 (0.80, 2.03) | 1.58 (0.93, 2.69) | 0.11 |  | 2.18 (1.01, 4.67) | 0.04 |  | 1.19 (0.69, 2.06) | 0.53 |  |  |
| Q4 | 1.34 (0.82, 2.19) | 2.27 (1.32, 3.89) | 0.004 |  | 2.22 (1.03, 4.82) | 0.04 |  | 1.52 (0.85, 2.70) | 0.15 |  | 0.44 |
|  |  |  |  |  |  |  |  |  |  |  |  |
| **IHC4 score** |  |  |  |  |  |  |  |  |  |  |  |
| Q1 | 1.00 (reference) | 1.00 (reference) |  |  | 1.00 (reference) |  |  | 1.00 (reference) |  |  |  |
| Q2 | 1.08 (0.67, 1.73) | 0.81 (0.47, 1.38) | 0.51 |  | 0.93 (0.41, 2.10) | 0.86 |  | 0.84 (0.48, 1.46) | 0.54 |  |  |
| Q3 | 0.95 (0.58, 1.54) | 1.23 (0.72, 2.08) | 0.43 |  | 1.26 (0.58, 2.75) | 0.56 |  | 0.81 (0.46, 1.44) | 0.48 |  |  |
| Q4 | 0.82 (0.49, 1.37) | 0.82 (0.49, 1.37) | 0.44 |  | 1.16 (0.52, 2.61) | 0.71 |  | 0.59 (0.33, 1.09) | 0.09 |  | 0.04 |
|  |  |  |  |  |  |  |  |  |  |  |  |

Odds ratios and 95% confidence intervals were from polytomous logistic regression models adjusted for age, parity, age at menarche, family history of breast cancer in a first degree relative, breast cancer subtype (in NPI model), and the NPI (in IHC4 score model).
